# The risk of materno-fetal infection. Importance of common laboratory tests

**DOI:** 10.1186/1471-2334-13-S1-P112

**Published:** 2013-12-16

**Authors:** Ioana Roşca, Violeta Oriță, Raluca Popescu, Marcela Șerban, Roxana Smadeanu, Mihai Mitran

**Affiliations:** 1 Clinical Hospital of Obstetrics and Gynecology “Prof. Dr. Panait Sârbu”, Bucharest, Romania; 2 “Grigore Alexandrescu” Clinical Emergency Hospital, Bucharest, Romania; 3 The Institute for Mother and Child Protection “Alfred Rusescu”, Bucharest, Romania

## Background

Maternal infection is an important cause of morbidity and mortality in newborns. Rupture of membranes more than 18 hours before birth, fever during labor, urinary tract infections or vaginal infections (eg *Streptococcus* group B), treated or not treated during pregnancy or labor, may be cause of serious illness in the newborn. The aim of this study was to observe the evolution of infants exposed to certain risk factors, using the total blood count and the determination of C-reactive protein.

## Methods

The authors have proposed a study on term babies born in our maternity “Prof. Dr. Panait Sârbu”, Bucharest, during 01 August 2012 – 01 August 2013, exposed to the following risk factors: rupture of membranes more than 18 hours before birth, vaginal infections treated or untreated during pregnancy, urinary infections, pregnant woman with fever during labor, using the total blood count and the value of C reactive protein.

## Results

All infants entered into the study were evaluated after at least 12 hours of life, determining the total blood count and C-reactive protein. There have been changes to these tests to a large number of subjects included in the study, and there have been cases in which, though the usual tests were within normal limits, infants developed symptoms, with no altered values of these laboratory tests (up to 72 hours). The infants exposed to certain risk factors (membranes ruptured more than 18 hours before birth and the presence of group B *Streptococcus* in maternal cultures, untreated antepartum) received prophylactic antibiotics at birth, however, registering changes in blood counts (leukocytosis/leukocytopenia) and/or increased levels of C-reactive protein in 10% of cases (resistance to antibiotics administered anterpartum/intrapartum?).

## Conclusion

The result of common tests can be used to evaluate the newborn exposed to certain infectious factors, and to specify the correct therapeutic attitude, but in close relation with the clinical outcome, considering their non-specificity and potential false positive or negative results.

